# Thermal Insulation Performance of SiC-Doped Silica Aerogels under Large Temperature and Air Pressure Differences

**DOI:** 10.3390/gels8050320

**Published:** 2022-05-20

**Authors:** Sheng-Nan Zhang, Hao-Qiang Pang, Ting-Hui Fan, Qing Ye, Qi-Lin Cai, Xi Wu

**Affiliations:** 1 College of Energy, Soochow University, 333 East Ganjiang Road, Suzhou 215031, China; zsnayuan@163.com (S.-N.Z.); 20215240009@stu.suda.edu.cn (T.-H.F.); yeqing@suda.edu.cn (Q.Y.); qlcai@suda.edu.cn (Q.-L.C.); 2 School of Materials Science and Engineering, Shanghai University, Shanghai 200444, China

**Keywords:** SiC-doped silica aerogel, thermal insulation performance, transient pressure change, large temperature difference

## Abstract

Silica aerogel composite is an excellent thermal insulator for spacecraft under high-temperature and complex air environments. This study intends to evaluate SiC-doped silica aerogel’s thermal insulation performance under large temperature and air pressure differences. In this paper, the hot surface’s temperature response of SiC-doped silica aerogel with different content was studied at significant temperature differences (Δ*T*) when pressure changes instantaneously. Their thermal insulation performance was evaluated by analyzing the influence of pressure gradients on the unsteady-state heat transfer. When the cold surface’s temperature of the specimen keeps constant at 15 °C and Δ*T* = 171~912 K, the results demonstrate that the correlative thermal conductivities of silica aerogel with 1% and 5.84% SiC are 0.02223~0.04077 W·m^−1^·K^−1^ at *P* ≈ 10 Pa and 0.03165~0.04665 W·m^−1^·K^−1^ at *P* = 1 atm, respectively. The aerogel composite with 0% SiC showed the best thermal insulation performance at Δ*T* < 200 K and *P* ≈ 10 Pa, while the aerogel with 5.84% SiC became the best at Δ*T* > 700 K and *P* = 1 atm. In addition, the transient pressure decreases will significantly impair the heat transfer of the gas inside the aerogel, thereby weakening the gaseous thermal conductivity and improving the thermal insulation performance.

## 1. Introduction

Silica aerogel is a typical nano-porous material [1] with the advantages of a significant specific surface area [2], high porosity [3], low density [4], and ultra-low thermal conductivity [5]. Aerogel’s ultra-low thermal conductivity, which mainly causes its excellent thermal insulation performance, is attributed to the complex nano-/micro-structure. The nanoparticles gather together randomly and form catenulate backbones, connecting and producing a three-dimensional network skeleton [6]. Such a skeleton creates large numbers of nanopores, which reduces the mean free path of the gas molecules, thus weakening the heat transfer between the gas molecules [7]. The heat conducted by gas molecules is much greater than in solid-phase, with 50–80% [8]. Kistler [9] first derived the relationship between the gaseous thermal conductivity and the gas molecules’ mean free path in aerogels from the perspective of molecular kinematics; Zeng [10] considered the collisions between gas molecules and solid walls inside nanopores to derive the gaseous thermal conductivity model. The above studies only revealed aerogel’s insulation mechanism of gaseous conduction. However, pure silica aerogel is almost transparent to infrared radiation with wavelengths between 3 and 8 μm [11]. Meanwhile, it has a large share of thermal radiation at high temperatures. So opacifier needs to be added to optimize its thermal insulation performance. The complex refractive index is an important parameter affecting optical performance. Its real part represents the refraction of electromagnetic waves by the medium, and the imaginary part stands for the absorption of electromagnetic waves by the medium, which means that the light in the medium is attenuated. Due to larger real and imaginary parts, SiC [12] has a good shading effect. Therefore, SiC, an effective opacifier, is doped into silica aerogels to restrain infrared radiation at large temperature differences (>100 K).

Silica aerogel’s excellent thermal insulation performance has attracted extraordinary attention in aerospace. It was first applied to the Mars Sojourner rover as an insulator to protect the primary battery from extremely low temperatures [13]. NASA had also utilized aerogel as a core material in Venus spacecraft’s thermal protection system [14], which experienced sudden changes during lift-off and re-entry into the atmosphere and thus caused significant temperature differences and air pressure fluctuations [15,16]. Silica aerogel, a thermal insulation material for spacecraft, is forced to put up with a high temperature (>1000 °C) and transient pressure changes. Thermal conductivity, predominantly gaseous thermal conductivity, is strongly influenced by the atmospheric environment (temperature and pressure). However, only a few studies are about aerogel’s thermal insulation performance variation under large temperature differences and transient pressure change, and the experimental investigation is even less.

Most current methods for measuring the thermal conductivity of aerogels are carried out in a static air pressure environment. Zeng [17] tested silica aerogel’s gaseous thermal conductivity at *T* = 296 K in air, which decreased sharply to 0 when *P* < 0.01 bar. Spagnol [18] measured silica aerogel’s thermal conductivity at *T* = 300~315 K under variable atmospheric pressure, which accounted for over 60% of its total thermal conductivity. Zhang’s experimental results [19] proved that gaseous thermal conductivity was strongly affected by gas pressure. Nevertheless, the above methods are generally less than the slight temperature difference, 5~50 K, demanded by the standards [20,21,22]. The wall-temperature response method [23,24] evaluates the thermal insulation performance of materials at a significant temperature difference. However, the specimen’s cold surface is usually exposed to the air environment and cannot be easily controlled and quantitatively determined, thus influencing the back-surface temperature response. Therefore, Pang et al. [25] proposed an experimental system to investigate silica aerogel composites’ thermal insulation performance by measuring the hot surface’s temperature response during the unsteady state and steady state at large temperature differences and standard atmospheric pressure.

Hence, it is necessary to study silica aerogel’s thermal insulation performance by the temperature response at pressure changes instantaneously and under large temperature difference conditions. In this paper, the hot surface’s temperature response of silica aerogel composites with different SiC components is measured under Δ*T* = 0–912 K and *P* = 0–1 atm; their thermal conductivities are further calculated. Then, their thermal insulation performance prediction model is proposed to describe unsteady-state heat transfer under the experiment’s same pressure and temperature conditions. Finally, the influence of pressure difference (Δ*P*) and porosity on silica aerogel’s thermal insulation is excavated.

## 2. Experiment

### 2.1. Experimental System

**Figure 1:**
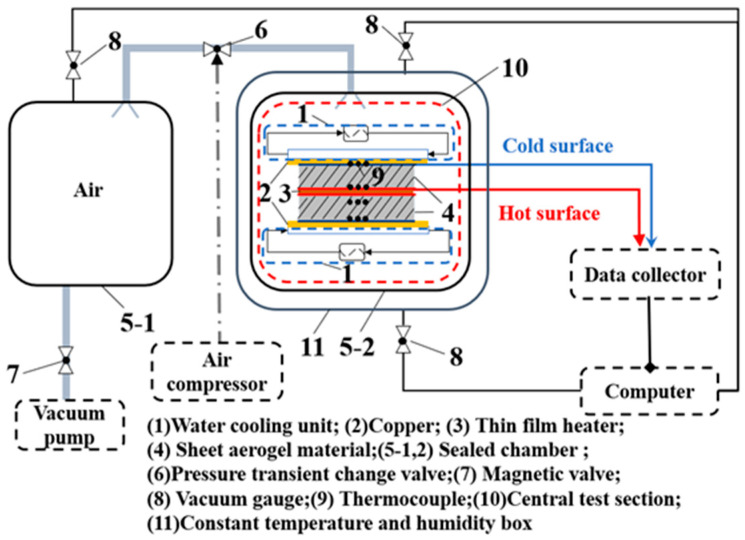
Experimental system.

An experimental system for testing the dynamic hot surface’s temperature response of monolithic silica aerogel is established, as shown in Figure 1. The system includes the central test section (Figure 1(10)), heating system, cooling system, variable atmospheric pressure system, data acquisition system, and constant temperature environment system. The central part of the system is a symmetrical sandwich structure with a thin film heater (Figure 1(3)) in the middle, which is sandwiched between the two hot surfaces of the upper and lower monolithic silica aerogel. The water-cooling unit is two identical copper sheets (Figure 1(2)) close to the cold surface to keep the specimen at the approved temperature. The variable atmospheric pressure system contains two sealed chambers (Figure 1(5-1, 2)), an air compressor, a constant temperature and humidity box (Figure 1(11)), a vacuum pump, and a pressure transient change valve (Figure 1(6)). The central test system is placed in a sealed chamber (Figure 1(5-2)), and the pressure change condition can be achieved by adjusting the pressure transient valve of the variable atmospheric pressure system.

The hot surface’s heat flux of the specimen keeps constant, and the temperature of the cold surface, initial moment, and environment stay constant at 288.15 K. The air pressure can be adjusted from 10 Pa to 1 bar, and the temperature difference is from 0 to 912 K. The air pressure will be changed instantaneously when the heat transfer reaches the first steady state. The heat transfer covers the initial steady state, first unsteady state when heated, first steady state, second unsteady state when pressure changes instantaneously, and second steady state in the experimental test process.

### 2.2. Material and Testing Procedure

The components and parameters of silica aerogel doping with 3.5 μm SiC are listed in Table 1, the volume fraction of which is 0, 1, and 5.84%, respectively.

The experimental procedures are introduced as follows:

(1) Dehydrate two identical samples in a drying oven to remove water vapor.
(2) Put the specimens sandwiched with the heater, thermocouples, and cooling units and place the main test section into the sealed chamber, as shown in Figure 1.
(3) Set the initial and ambient temperature identical and run the system.
(4) Turn on the D.C. power supply when the specimen’s heat transfer reaches the initial steady state; change the atmospheric pressure through the pressure transient valve at the first steady state.
(5) Record the experimental data of the whole process by the thermocouples. Shut down the test system after the second steady state.

## 3. Theoretical Analysis Model

### 3.1. Thermal Conductivity

**Figure 2:**
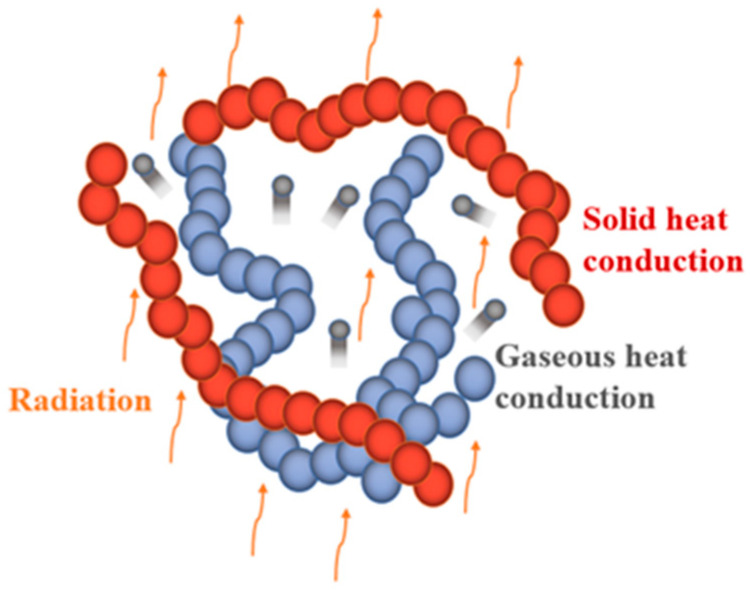
Heat transfer inside silica aerogel.

The heat transfer process inside the silica aerogel usually consists of three parts [26,27], as shown in Figure 2: heat conduction through the solid skeleton, heat transfer through the gas molecules, and heat transfer by radiation.

The gaseous thermal conductivity model of silica aerogel can be described as follows [10,27]:

(1) $$\lambda_{g}(P,T)=\frac{60.22\times PT^{-0.5}}{0.25S_{s}\rho \varphi^{-1}+4.01\times 10^{4}\times PT^{-1}}$$

where *P* is pressure; *T* represents temperature; *S*_s_ = (324.3/*ρ* + 5.03) × 10^5^ denotes specific surface area [28]; *ρ* stands for the density of the specimen in Table 1; *φ* means porosity in Table 1.

The solid thermal conductivity of bulk silicon dioxide is written as [29]:

(2) $$\lambda_{\mathrm{bulk}}=7.5264\times 10^{-1}+3.13\times 10^{-3}T-4.5242\times 10^{-6}T^{2}+3.5253\times 10^{-9}T^{3}$$

Silica aerogel’s solid thermal conductivity can be expressed as [30]:

(3) $$\lambda_{s}(P,T)=\frac{3r^{*}/4}{3r^{*}/4+1}\lambda_{\mathrm{bulk}}$$

where r* = *r*/*l* means the nondimensional radius of particles; *l* = 0.588 nm [31] represents phonon mean free path of amorphous; *r* = 3 nm [28] is radius of particles.

SiC is randomly distributed in the silica aerogel. Thus, SiC-doped silica aerogel’s effective thermal conductivity, *λ*_e_, is formed by a two-phase system described by the Maxwell model [32]:

(4) $$\frac{\lambda_{e}(P,T)}{\lambda_{a}(P,T)}=1+\frac{3(\lambda_{f}/\lambda_{a}(P,T)-1)f_{v}}{(\lambda_{f}/\lambda_{a}(P,T)+2)-(\lambda_{f}/\lambda_{a}(P,T)-1)f_{v}}$$

where *λ*_a_(*P*, *T*) = *λ*_g_(*P*, *T*) + *λ*_s_(*P*, *T*) is the pure silica aerogel’s effective thermal conductivity contributed by heat conduction; *λ*_f_ is the thermal conductivity of the SiC [33]; *f*_v_ is the volume fraction of the additive.

The radiative thermal conductivity of aerogels can be calculated using the Rosseland equation [34]:

(5) $$\lambda_{r}(P,T)=\frac{16\sigma n^{2}T^{3}}{3\sigma_{\mathrm{eR}}}$$

where *σ* is the Stefan-Boltzmann constant; *n* means the ambient medium’s refractive index; *σ*_eR_ is related to the spectral extinction coefficient of the material [35,36].

Hence, the effective thermal conductivity of silica aerogel can be calculated as:

(6) $$\lambda \left(P,T\right)=\lambda_{e}\left(P,T\right)+\lambda_{r}\left(P,T\right)$$

### 3.2. 3-D Unsteady-State Heat Transfer Model

**Figure 3:**
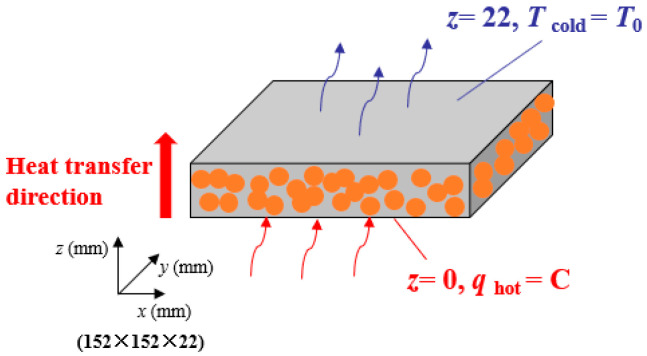
3-D unsteady heat transfer model.

The central test section is a symmetrical sandwich structure, and Ref. [25] has proved that a one-dimensional (1-D) heat transfer area exists in the center of the main test section, and heat transfers symmetrically up and down along the central axis. Therefore, Figure 3 displays a 3-D unsteady-state heat transfer model of SiC-doped silica aerogel.

The energy equation can be expressed as:

(7) $$\frac{\partial (\rho c_{p}T)}{\partial_{t}}=\lambda (P,T)\frac{\partial^{2}T}{\partial x^{2}}+\lambda (P,T)\frac{\partial^{2}T}{\partial y^{2}}+\lambda (P,T)\frac{\partial^{2}T}{\partial z^{2}}-\frac{\partial q_{r}}{\partial x}-\frac{\partial q_{r}}{\partial y}-\frac{\partial q_{r}}{\partial z}$$

where *c*_p_ represents the specific heat capacity of silica aerogel [37]; *t* reflects the time; *x*, *y*, and *z* candidates the geometric coordinate direction; *q*_r_ means radiative heat flux, described by Rosseland diffusion approximation [38].

The initial and boundary conditions can be expressed as:

(8) $$\left\{\begin{array}{cc} T=T_{0},P=P_{0}; & t=0\\ -\lambda (P,T)\frac{\partial T_{\mathrm{hot}}}{\partial x}=q_{\mathrm{hot}},P_{\mathrm{hot}}=P_{0}; & z=0,0<t<\tau \\ T_{\mathrm{cold}}=T_{0},P_{\mathrm{cold}}=P_{0}; & z=\delta ,0<t<\tau \end{array}\right.$$

where *T*_0_ and *P*_0_ represent the initial temperature and pressure, respectively; *T*_hot_, *T*_cold_, *P*_hot_, and *P*_cold_ are the temperature of the specimen’s hot surface and cold surface and the pressure of the specimen’s hot surface and cold surface, respectively; *τ* is the moment when the pressure changes instantaneously; *δ* = 22 mm reflects the thickness of the specimen; *λ*(*P*, *T*) is the thermal conductivity of the aerogel in Equation (6).

The energy and pressure equations above are coupled and solved discretely using the finite volume method (FVM) [39]. The steps are as follows: First, input the geometric and physical parameters. The mesh generation of the computational region is based on the Cell Center Scheme. Then, initialize the temperature conditions. Next, the temperature field is calculated from the energy equation (Equation (7)). Finally, the hot surface’s temperature response is obtained.

## 4. Results and Discussion

### 4.1. Thermal Insulation Performance by Experiment

**Figure 4:**
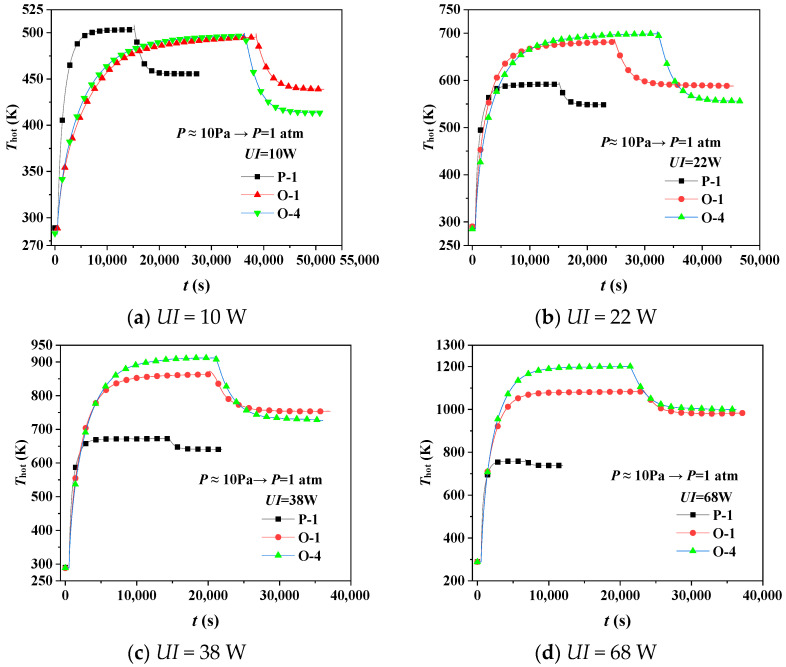
Hot surface’s temperature response of aerogels before and after pressure transient changes at Δ*T* = 0~912 K.

Figure 4a–d reveals the hot surface’s temperature response at *UI* = 10~68 W, respectively, and atmospheric pressure changes abruptly from *P* ≈ 10 Pa to *P* = 1 atm. Qualitatively, the higher the hot surface’s temperature is, and the faster *T*_hot_ rises, the better the thermal insulation performance has.

In Figure 4a, specimen P−1′s *T*_hot_ responds the fastest, meaning aerogel with no SiC additive has the best insulation performance at *UI* = 10 W. However, the *T*_hot_ of specimen P-1 becomes lower than specimens O−1 and O−4 in Figure 4b–d, which means insulation performance of aerogel with SiC becomes better than aerogel with no SiC with the heating power increases. It reveals that SiC could restrain thermal radiation effectively at high temperatures. Furthermore, focusing on the *T*_hot_ of specimens O−1 and O−4 both at *P* ≈ 10 Pa and *P* = 1 atm, the deviation of *T*_hot_ between specimens O−1 and O−4 becomes more significant as heating power increases, which means specimen O−4′s insulation performance becomes better than that of specimen O−1. SiC particles, which are added to silica aerogel, increase the contribution of the solid heat conduction and decrease the thermal radiation’s contribution. The heat conduction is prominent when the temperature difference is relatively small, while the thermal radiation becomes dominant at high temperatures.

Further, specimen O−4′s Δ*T* between the *T*_hot_ at the first and second steady state is more significant than that of specimen O−1 before and after the transient pressure changes. The porosity of specimen O−4 is more significant than that of specimen O−1, and thus gaseous heat conduction’s contribution of specimen O−4 is more extensive than that of specimen O−1 as heating power increases. It is because air entry increases the contribution of the gaseous heat conduction after the transient pressure change.

### 4.2. Correlative Thermal Conductivity of SiC-Doped Silica Aerogel

Quantitatively, the thermal insulation performance of SiC-doped silica aerogel is evaluated by the correlative thermal conductivity in the first and second steady state. Note that such correlative thermal conductivity at large temperature differences is not defined in thermal physics but can evaluate the specimen’s thermal insulation performance [25].

(9) $$\bar{\lambda }(P,T)=\frac{UI}{2A}\cdot \frac{\delta }{T_{\mathrm{hot}}-T_{\mathrm{cold}}}$$

where $\;\bar{\lambda }(P,\;T)$ is the equivalent thermal conductivity of the silica aerogel; *U* refers to the voltage; *I* represents the electric current; *A* is the cross-section area.

To excavate the effect of SiC at a different temperature, we calculated the correlative thermal conductivity of specimens P−1, O−1, and O−4 in Figure 4, respectively, as shown in Table 2. The correlative thermal conductivity of specimens P−1, O−1, and O−4 equal 0.02220~0.06901, 0.02304~0.04077, and 0.02223~0.03550 W·m^−1^·K^−1^ at the first steady state, and 0.02856~0.07257, 0.03165~0.04665, and 0.03653~0.04541 W·m^−1^·K^−1^ at the second steady state.

The correlative thermal conductivity of specimen P-1 is consistently lower than that of specimen O−1 and specimen O−4 at Δ*T* < 214 K because SiC as a solid skeleton greatly influences increasing solid thermal conductivity, while the suppressive effect on radiative heat transfer is almost non-existent at low Δ*T*. With Δ*T* increases, the correlative thermal conductivity of specimen O−1 and specimen O−4 becomes larger than that of specimen P−1, which means that SiC, owning excellent light absorption and light refraction performance, can effectively suppress infrared radiation at large temperature differences. At Δ*T* > 713 K and *P* = 1 atm, the thermal conductivity of specimen O−4 exceeds that of specimen O−1, implying that the thermal insulation of silica aerogel with more SiC becomes better than that of silica aerogel with less SiC. The reason is that the effect of radiative heat transfer on the thermal insulation performance becomes more and more significant as Δ*T* becomes larger.

### 4.3. Hot Surface’s Temperature Variation by Simulation at Transient Pressure Change

**Figure 5:**
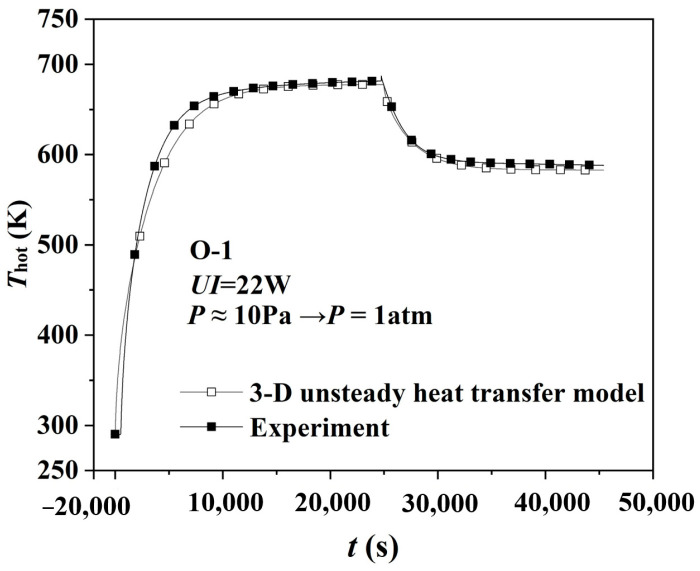
Comparison of model simulation and experimental data of specimen O−1.

Limited by the experimental conditions, we only tested the hot surface’s temperature response at transient pressure increase. Therefore, this part further evaluates SiC-doped silica aerogel’s thermal insulation performance under different transient pressure change conditions, including transient pressure decrease. Figure 5 compares the hot surface’s temperature response of the specimen O−1 by Equation (7) and the experiment before and after transient pressure decrease, and the maximum deviation is no more than 3.26%. Therefore, the 3-D unsteady-state heat transfer model is reliable.

**Figure 6:**
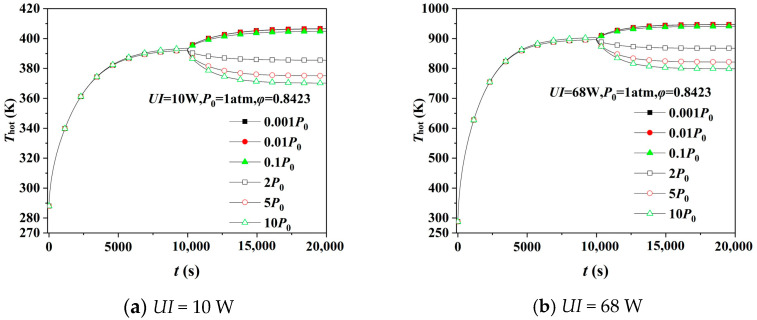
Simulation of hot surface’s temperature response of aerogel under different transient pressures.

Figure 6 depicts the hot surface’s temperature response of the silica aerogel simulated by the heat transfer model, and the pressure conditions include transient pressure increases/decreases and multiple pressure differences. The degree of variation in *T*_hot_ increases with the increasing Δ*P* when transient pressure rises, so the more discrepancy the insulation performance will be after pressure increases. The reason is that the movement of the gas molecules becomes violent as Δ*P* increases, and thus the insulation performance deteriorates. When transient pressure decreases, *T*_hot_ will increase because the amount of gas molecules inside decreases, and the gas molecules’ movement is weakened, enhancing thermal insulation performance. However, the diversity of *T*_hot_ between different Δ*P* is tiny because the gaseous thermal conductivity decreases almost the same when *P* < 0.1 atm [10].

To better visualize the effect of transient pressure changes, we calculated the correlative thermal conductivity of the aerogel and its change rate at a steady state before and after the instantaneous pressure change in Figure 6b. The relevant results are listed in Table 3. The thermal conductivity changed from 0.05361 to 0.04958, 0.04916, and 0.04911 W·m^−1^·K^−1^ as the pressure dropped instantaneously from *P*_0_ to 0.1 *P*_0_, 0.01 P_0_, and 0.001 *P*_0_. The thermal conductivity decreased by 7.52, 8.30, and 8.39% after the pressure drop. The Knudsen effect reduces the gaseous thermal conductivity in small confined spaces. As the pressure decreases, the mean free path of the gas molecules decreases, resulting in a more substantial Knudsen effect and lower thermal conductivity of the gas in porous structures. Meanwhile, the gaseous thermal conductivity effect is extremely limited as the gas inside the pores becomes thinner. Therefore, the difference in thermal conductivity at different pressure drops is insignificant.

More intense heat transfer due to the more violent collisions between gas molecules after the transient pressure rise, so the thermal conductivity becomes 0.05587, 0.06069, and 0.06336 W·m^−1^·K^−1^, an increase of 4.22, 13.21, and 18.19%, respectively.

### 4.4. Hot Surface’s Temperature at Different Porosity

**Figure 7:**
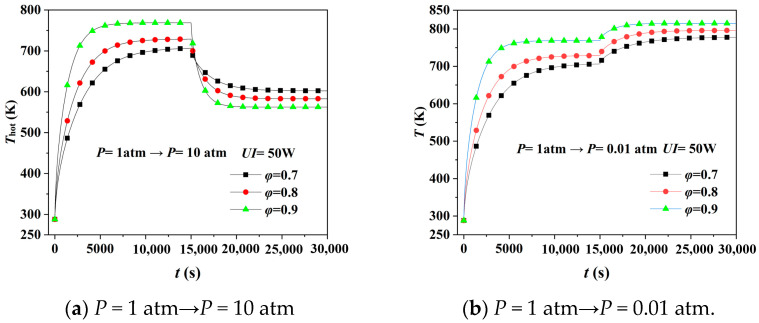
Simulation of hot surface’s temperature response of aerogel with different porosity under transient pressure change.

Figure 7a,b demonstrate the hot surface’s temperature response of aerogel with different porosity at transient pressure increases and decreases. Figure 7a represents that the aerogel with a porosity of 0.9 has the best thermal insulation performance before the transient pressure increases, while the worst after that. The gas molecules inside the aerogel are in violent motion when pressure increases sharply, and thus aerogel with more significant porosity has a more extensive deviation of the thermal insulation performance after transient pressure increases. When the pressure transient decreases in Figure 7b, the gas within the aerogel becomes rarefied at *P* < 0.01 atm, making the gaseous thermal conductivity almost ineffective, so *T*_hot_ rises, but the difference is tiny. Heat transfer mainly relies on the aerogel’s solid skeleton at this point so that aerogel with more significant porosity has a better thermal insulation performance.

Table 4 shows the correlative thermal conductivity and change rate at a steady state calculated based on Figure 7. More frequent collisions between molecules and more significant heat transfer due to more gas molecules in the pores, so the increase rate of thermal conductivity after transient pressure rise becomes from 33.10%, 49.13% to 75.12% with the increase of porosity. On the other hand, since the number of gas molecules decreases sharply after the transient pressure drop, the gas-contributed thermal conductivity is significantly reduced. Aerogel with the highest porosity (0.9) has the lowest thermal conductivity (0.04518 W·m^−1^·K^−1^) and the best thermal insulation.

## 5. Conclusions

The thermal insulation performance of SiC-doped silica aerogel is investigated by testing their hot surface’s temperature response at transient pressure changes and large temperature differences. The insulation performance prediction model is proposed to describe unsteady-state heat transfer. Furthermore, the influence of the critical parameters on the thermal insulation is excavated based on the prediction model. The conclusions are drawn as follows:

(1) Transient pressure changes the heat transfer of the gas inside the aerogel, affecting the gaseous thermal conductivity and the thermal insulation performance. When the pressure decreases instantaneously, the hot surface’s temperature increases, and its thermal insulation performance is improved, and vice versa. The more extensive the transient pressure change is, the more significant the thermal insulation performance’s variation will be.
(2) When *T*_cold_ = 15 °C and Δ*T* = 171~912 K, the effective thermal conductivities of SiC-doped silica aerogel with 0, 1, and 5.84% SiC are 0.02220~0.06901, 0.02304~0.04077, and 0.02228~0.03550 W·m^−1^·K^−1^ at *P* ≈ 10 Pa, and 0.02856~0.07207, 0.03165~0.04665, and 0.03653~0.04541 W·m^−1^·K^−1^ at *P* = 1 atm, respectively.
(3) The higher SiC content increases the solid thermal conductivity of the aerogel, but the ability to restrain thermal radiation is more significant at large temperature differences.
(4) Aerogels with greater porosity are strongly influenced by the movement of the internal gas molecules at transient pressure changes. The larger the porosity of the aerogel, the greater the influence of the internal molecular motion, the more noticeable change of the gaseous thermal conductivity, and the more significant change in thermal insulation performance.

## Figures and Tables

**Table 1:** Components and parameters of silica aerogel specimen.

No.	Matrix Silica Aerogel (*f*_v_, %)	SiC (*f*_v_, %)	Porosity (*φ*, %)	Density (*ρ*, kg·m^−3^)
P−1	99.49	0	89.87	346.39
O−1	98.49	1	84.23	354.06
O−4	93.65	5.84	88.57	387.14

**Table 2:** Correlative thermal conductivity of specimens P−1, O−1, and O−4.

*P*/W	Correlative Thermal Conductivity/(W·m^−1^·K^−1^)											
	P−1				O−1				O−4			
	Δ*T* /K	Steady State 1st	Δ*T* /K	Steady State 2nd	Δ*T* /K	Steady State 1st	Δ*T* /K	Steady State 2nd	Δ /K	Steady State 1st	Δ*T* /K	Steady State 2nd
10	214.33	0.02220	455.57	0.02856	171.09	0.02304	150.92	0.03165	208.54	0.02223	125.46	0.03653
22	303.92	0.03459	548.40	0.04040	398.53	0.02643	300.13	0.03515	410.81	0.02532	267.97	0.03867
38	384.53	0.04726	640.51	0.05158	585.01	0.03091	465.75	0.03882	624.01	0.02901	438.56	0.04129
68	470.12	0.06901	738.23	0.07207	759.16	0.04077	695.02	0.04665	912.37	0.03550	713.30	0.04541

**Table 3:** Correlative thermal conductivity of silica aerogel at different transient pressures and *UI =* 68 W.

**Pressure/(atm)**	***P*_0_**	**0.1*P*_0_**	**0.01*P*_0_**	**0.001*P*_0_**	**2*P*_0_**	**5*P*_0_**	**10*P*_0_**
$\bar{\lambda }$/(W·m^−1^·K^−1^)	0.05361	0.04958	0.04916	0.04911	0.05587	0.06069	0.06336
Change rate/(%)	/	−7.52	−8.30	−8.39	+4.22	+13.21	+18.19

**Table 4:** Correlative thermal conductivity of silica aerogel with different porosity at *UI =* 50 W.

Porosity	*P*_0_	10*P*_0_		0.01*P*_0_	
	$\bar{\lambda }$/(W·m^−1^·K^−1^)	$\bar{\lambda }$/(W·m^−1^·K^−1^)	Change Rate/(%)	$\bar{\lambda }$/(W·m^−1^·K^−1^)	Change Rate/(%)
0.7	0.05691	0.07575	+33.10	0.04863	−14.55
0.8	0.05402	0.08072	+49.43	0.04687	−13.24
0.9	0.04951	0.08670	+75.12	0.04518	−8.75

## Data Availability

This study did not report any data (All the data is included in the current manuscript).
